# A Systematic Review of Systemic Treatment Options for Advanced Non-Clear Cell Renal Cell Carcinoma

**DOI:** 10.3233/kca-190078

**Published:** 2020-03-30

**Authors:** Chelsea K. Osterman, Tracy L. Rose

**Affiliations:** a Division of Hematology/Oncology, University of North Carolina, Chapel Hill, NC, USA; b Division of Hematology/Oncology, Lineberger Comprehensive Cancer Center, University of North Carolina, Chapel Hill, NC, USA

**Keywords:** Renal cell carcinoma, non-clear cell, papillary, chromophobe, collecting duct, metastatic, systemic treatment

## Abstract

**Introduction:**

There have been a number of recent advances in the management of advanced clear cell renal cell carcinoma (ccRCC). However, the majority of these studies excluded patients with non-clear cell RCC (nccRCC), and optimal management of nccRCC remains unknown.

**Materials and Methods:**

A systematic review of the literature was performed according to the Preferred Reporting Items for Systematic Reviews and Meta-Analyses (PRISMA) guidelines to evaluate systemic treatment options in locally advanced or metastatic nccRCC between 2000-2019. Randomized controlled trials, single-arm phase II–IV trials, and prospective analyses of medication access programs were included. The primary outcome measures were progression free survival (PFS), overall survival (OS), and objective response rate (ORR).

**Results:**

A total of 31 studies were included in the final analysis. There was the highest level of evidence to support first-line treatment of nccRCC with sunitinib. Additional single-arm trials support the use of other vascular endothelial growth factor (VEGF) inhibitors with axitinib and pazopanib, as well as mammalian target of rapamycin (mTOR) inhibition with temsirolimus or everolimus +/− bevacizumab. Immune checkpoint inhibition has an emerging role in nccRCC, but optimal sequencing of available options is not clear. Prospective data to support the use of newer immunotherapy combinations are lacking. Treatment for collecting duct carcinoma remains platinum-based chemotherapy.

**Conclusions:**

The availability of randomized trials in nccRCC is limited, and most studies include outcomes for nccRCC as a group, making conclusions about efficacy by subtype difficult. This systematic review supports consensus guidelines recommending sunitinib or clinical trial enrollment as preferred first-line treatment options for nccRCC, but also suggests a more nuanced approach to management and new options for therapy such as immune checkpoint inhibition.

## INTRODUCTION

Cancers of the kidney and renal pelvis account for about 4% of all new cancer diagnoses per year in the US with an estimated 73,820 new diagnoses in 2019 [1]. The vast majority of these are renal cell carcinomas (RCC) with clear cell renal cell carcinoma (ccRCC) as the most common subtype, comprising 75–80% of all RCC cases [2]. The remainder of cases are classified as non-clear cell renal cell carcinoma (nccRCC), which are then divided into multiple distinct subtypes based on histological and molecular characteristics. Subtypes of nccRCC include papillary, chromophobe, collecting duct, renal medullary, and translocation RCC, which represent 10–15%, 5–7%, 1–2%, <1%, and < 1% of all RCCs, respectively [3]. Unclassifiable cases of RCC are also typically included under the nccRCC umbrella, and both ccRCC and nccRCC can have sarcomatoid differentiation.

Median survival of patients with localized nccRCC varies with histology, with more favorable outcomes in patients with papillary and chromophobe RCC and less favorable outcomes in patients with renal medullary and translocation RCC [4]. In the metastatic setting, however, survival in all subtypes of nccRCC is uniformly worse compared to ccRCC [5], due to the inherent aggressiveness of these cancers, and a lack of effective systemic treatment options. Median survival following a diagnosis of metastatic nccRCC remains poor with 5 year overall survival rates of 7–12% [6].

Recently, there have been a number of promising advances in the treatment of metastatic ccRCC, particularly with immune checkpoint inhibitors (ICIs) and novel tyrosine kinase inhibitors (TKIs) [7-10]. These clinical trials have generally excluded patients with nccRCC and so data to support the use of these newer agents in the nccRCC population are lacking. To date, there are only 3 randomized controlled trials (RCTs) that exclusively enrolled nccRCC patients and another 2 RCTs that stratified results by histology [11-15]. However, there are a number of single-arm trials and prospective analyses of expanded access programs that evaluate additional therapeutic options for nccRCC patients and can provide valuable information for this under-represented cohort.

The goal of this systematic review was to evaluate the existing prospective literature regarding systemic treatment of advanced or metastatic nccRCC. In particular, we sought to highlight new agents and combinations that show potential, and to compile the existing evidence base for treatment stratified by nccRCC histologic subtype.

## MATERIALS AND METHODS

### Search strategy

A systematic review of the literature was performed according to the Preferred Reporting Items for Systematic Reviews and Meta-Analyses protocol [16] to identify studies evaluating systemic treatment options in locally advanced or metastatic nccRCC. Study selection was performed in duplicate by C.O. and T.R. The PubMed-Medline and Embase databases were searched for studies published between January, 2000 and June, 2019 using one or a combination of the following search terms: renal cell carcinoma (RCC), advanced, metastatic, non-clear cell renal cell carcinoma, papillary RCC, chromophobe RCC, collecting duct RCC, translocation RCC, medullary RCC, systemic treatment, and clinical trial. Abstracts from the 2019 American Society of Clinical Oncology (ASCO) Annual Meeting and Genitourinary Cancer Symposium, and references found in relevant publications were also evaluated for inclusion. Results were restricted to English language only.

Study title and abstract were screened to determine initial relevance. Eligible articles then underwent full text evaluation for final inclusion in this review. Studies included were RCTs, single-arm phase II–IV trials, and prospective analyses of expanded access programs, while phase I trials, retrospective analyses, case series, case reports, meta-analyses, and reviews were excluded. If there were multiple publications reporting on the same cohort, only the most recent publication was included to avoid over-representation. Studies that did not report results for nccRCC patients alone, included less than 10 nccRCC patients, or evaluated surgical or radiation therapy were excluded.

### Data extraction

A data extraction form was generated and included study design, baseline patient characteristics including histology, intervention(s), and outcome measures. Data extraction was performed independently by C.O.

### Outcome measures

The primary outcome measures were progression free survival (PFS), overall survival (OS), and objective response rate (ORR). Due to the heterogeneous populations and methodologies of the included studies, data were not pooled for meta-analysis.

### Risk of bias assessment

The Cochrane Collaboration tool was used to assess risk of bias in RCTs [17].

## RESULTS

**Fig. 1.**
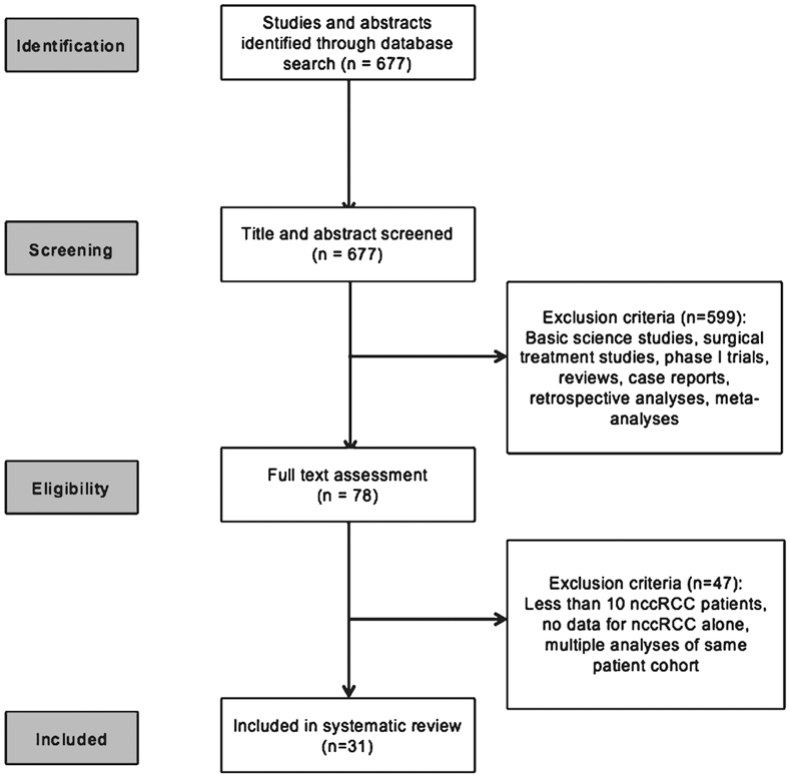
CONSORT diagram outlining the study evaluation and selection process.

The systematic search strategy identified 677 publications for screening. Of these, 78 studies underwent full text assessment and a total of 31 were included in the final systematic review (Fig. 1).

### Characteristics of included studies

The included studies were comprised of 5 RCTs, 1 single-arm phase IIIB/IV trial, 21 single-arm phase II trials, and 4 prospective analyses of expanded access medication programs. A total of 22 different systemic treatments for locally advanced or metastatic nccRCC were evaluated across a combined total of 2,134 nccRCC patients. Study characteristics and outcomes for all included studies are detailed in Tables 1-4 and supplementary Table 1.

### Risk of bias assessment of included studies

**Fig. 2.**
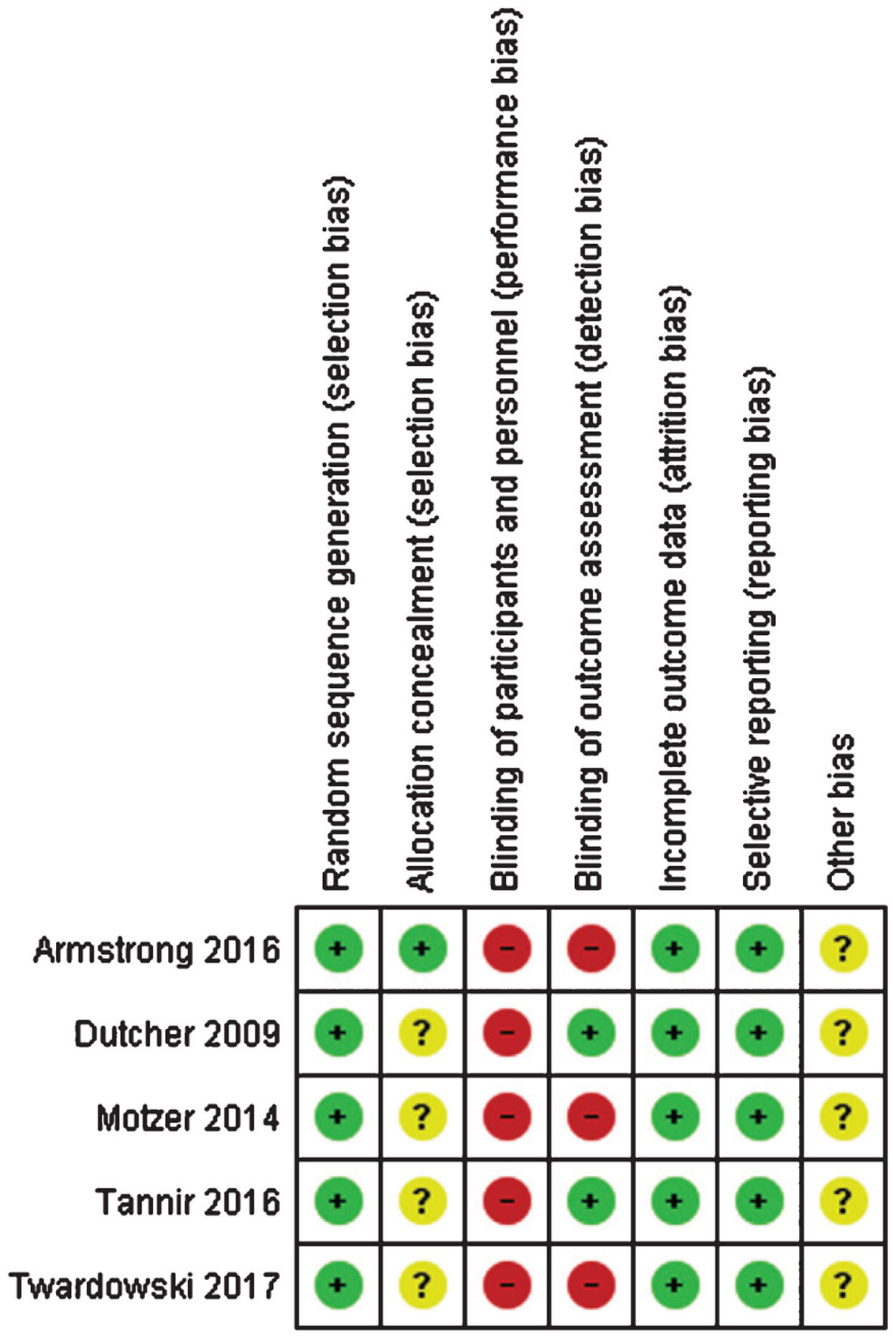
Risk of bias assessment of the randomized controlled trials included in the systematic review. Green (+): low risk of bias; yellow (?): unclear risk of bias; red (−): high risk of bias.

All 5 RCTs had a low overall risk of bias, although all of them were open-label and only 2 of the 5 trials included blinded independent review for outcome assessment (Fig. 2). The remaining single arm studies and expanded access programs had at least a moderate risk of bias, however they were still included in this systematic review as they represent much of the best available evidence for treatment in this patient population. Based on the inclusion of multiple negative studies within this review, we do not suspect that publication bias had a significant impact on our results or conclusions.

## RCTs in nccRCC

### Everolimus versus sunitinib

There were 3 RCTs comparing the mammalian target of rapamycin (mTOR) inhibitor everolimus to the vascular endothelial growth factor (VEGF) TKI sunitinib in first line treatment of metastatic nccRCC. The ASPEN and ESPN trials enrolled only nccRCC, and the RECORD-3 trial enrolled patients with any RCC histology but reported PFS results for nccRCC alone [11, 12, 15]. Median overall survival was numerically greater in the sunitinib group compared to the everolimus group in both ASPEN (31.5 months vs. 13.2 months; HR 1.12 (95% CI 0.7–2.1)) and ESPN (16.2 months vs. 14.9 months; stratified log-rank *p* = 0.18), however this failed to reach statistical significance in either trial. The median PFS was numerically longer with first line sunitinib compared to everolimus in all 3 trials, but was only statistically significant in the ASPEN (8.3 months vs. 5.6 months; HR 1.41 (80% CI 1.03–1.92)) and RECORD-3 (7.2 months vs. 5.1 months; HR 1.5 (95% CI 0.9–2.8)) trials. Response rates were reported in ASPEN and ESPN with higher ORR seen for the sunitinib group in both trials (18% vs. 9% and 9% vs. 3%, respectively).

### Interferon-alpha (IFNα) versus temsirolimus

The phase 3 Advanced Renal Cell Carcinoma (ARCC) trial randomized patients with poor risk RCC of any histology to treatment with the mTOR inhibitor temsirolimus or interferon-α (IFNα). The study subsequently performed an exploratory subgroup analysis of outcomes for nccRCC patients alone [14]. In nccRCC patients, median OS and PFS were significantly longer in the temsirolimus group compared to the IFNα group (11.6 months vs. 4.3 months; HR 0.49 (95% CI 0.29–0.85) and 7.0 months vs. 1.8 months; HR 0.38 (95% CI 0.23–0.62)), respectively. Response rates were not different between groups. Clinical benefit, defined as complete response (CR) plus partial response (PR) plus stable disease (SD), was reported in 15/37 (41%) temsirolimus patients and 3/36 (8%) IFNα patients (*p* = 0.002).

### Tivantinib versus tivantinib plus erlotinib

The Southwest Oncology Group (SWOG) 1107 trial compared the VEGF TKI tivantinib with or without the epidermal growth factor receptor (EGFR) TKI erlotinib in the first or second line setting for papillary RCC [13]. Unfortunately, the ORR was 0% in both arms and median OS and PFS were not different between the two arms.

## SINGLE-ARM TRIALS AND PROSPECTIVE ANALYSES OF EXPANDED ACCESS PROGRAMS IN nccRCC

### Anti-angiogenesis agents/Tyrosine kinase inhibitors

The majority of the single arm studies involving nccRCC patients evaluated TKIs targeting the VEGF pathway, including sunitinib [18-21], sorafenib [22-24], axitinib [25], and pazopanib [26].

### Sunitinib

Three single-arm studies of sunitinib enrolled only nccRCC patients with a total accrual of 111 patients, and a global expanded access program of sunitinib analyzed an additional 532 nccRCC patients. All four studies reported ORR (range 4.5–35.5%) and median PFS (range 2.7–6.4 months). Median OS was reported in two studies and ranged from 12.2–16.8 months.

### Sorafenib

Two single-arm studies and 1 expanded access program reported response rates to sorafenib for nccRCC patients. Khaled et al evaluated sorafenib in the first line setting and found a disease control rate (CR + PR + SD) of 81.8% for nccRCC patients, but ORR was not reported. In the second line setting, Procopio et al reported 1 papillary RCC patient with a partial response to sorafenib out of 18 total nccRCC patients (PR rate 5.6%). The expanded access trial by Stadler et al reported 4 partial responses out of 127 papillary or chromophobe RCC patients treated with sorafenib (ORR rate 3.1%). None of these studies reported OS or PFS results for nccRCC patients alone.

### Axitinib

One single-arm trial investigated axitinib in 40 nccRCC patients who had failed prior treatment with temsirolimus. The median OS, PFS, and ORR of the entire cohort were 12.1 months (95% CI 6.4–17.7), 7.4 months (95% CI 5.2–9.5), and 37.5%, respectively. Results were also reported by histology, with a median OS of 8.3 months (95% CI 4.1–12.5) and PFS of 3.5 months (95% CI 0–10.9) for papillary RCC, 22.2 months and 11.0 months for chromophobe RCC, and 16.9 months and 11.1 months (95% CI 7.6–14.6) for MiT family translocation RCC.

### Pazopanib

Pazopanib was evaluated in 29 nccRCC patients, primarily with papillary histology (65.5%). The ORR was 28% with a median PFS of 16.5 months (95% CI 10.9–22.1). Median OS was not reached, but the 1 year overall survival rate was 69%.

### mTOR inhibitors

In addition to the previously mentioned RCTs, there was one phase II trial of everolimus in nccRCC patients [27]. In this trial, the median OS of the entire cohort of 49 patients was 14.0 months with a PFS of 5.2 months and ORR of 10%. There was a trend toward increased PFS in patients with chromophobe RCC compared to papillary RCC (13.1 months vs. 3.4 months, *p* = 0.08), but no significant difference in OS (21.6 months vs. 10.9 months, *p* = 0.39)(27).

### mTOR inhibitors + bevacizumab

Two phase II trials evaluated the use of the angiogenesis inhibitor bevacizumab in combination with mTOR inhibition. A first-line trial of everolimus plus bevacizumab in nccRCC demonstrated a promising ORR of 26% [28]. In this trial, there were significant differences in outcomes based on histology, with the presence of papillary features associated with improved response. Compared to tumors without papillary features, those with papillary features had an increased ORR (43% versus 11%), PFS (12.9 months vs. 1.9 months), and OS (28.2 months vs. 9.3 months, *p* < 0.001). Furthermore, tumor genetic testing found mutations in *ARID1A* in 5 of 14 patients with a major papillary component but in none of the other histologic variants, and all 5 of these patients had a PFS > 6 months.

A trial of 40 RCC patients, including 13 with nccRCC, evaluated the combination of temsirolimus plus bevacizumab in patients that had disease progression or intolerable toxicity with a VEGF TKI [29]. Among patients with nccRCC, the ORR was 8%, although an additional 77% of patients had stable disease. Median OS was 13.1 months (95% CI 5.0–24.6) and median PFS was 5.6 months (95% CI 3.4–13.7).

### Immune checkpoint inhibitors

More recently, the safety and efficacy of immune checkpoint inhibitors (ICIs) has been explored in nccRCC through the KEYNOTE-427 study of pembrolizumab, a subgroup analyses of the CheckMate 374 study of nivolumab, and an expanded access program for nivolumab [30-32]. Additionally, a phase II trial of atezolizumab and bevacizumab included patients with nccRCC and clear cell renal cell carcinoma with sarcomatoid differentiation (sccRCC) [33].

Cohort B of the KEYNOTE-427 study [30] was the largest of the ICI studies and included 165 patients with nccRCC, the majority of which had papillary RCC (71.5%). One year PFS and OS rates were 24.7% and 73.7%, respectively. The ORR in the entire nccRCC cohort was 26.1%, including 6.1% of patients achieving a CR. ORR varied by histology, with an ORR of 28.0% for papillary RCC, 9.5% for chromophobe RCC, and 30.8% for unclassified RCC.

Subgroup analyses of CheckMate 374 [31] and an expanded access program of nivolumab [32] both showed activity in nccRCC, with an ORR of 13% and 19%, respectively. CheckMate 374 also reported a median PFS of 2.2 months (95% CI 1.8–5.4) and OS of 16.3 months (95% CI 9.2-NR). Additionally, a trial of atezolizumab plus bevacizumab had an ORR of 26% in patients with nccRCC, with survival data not yet mature [33].

Three of the four studies of ICIs included subgroup analysis of patients by PD-L1 status, all showing a numerically increased response rate in PD-L1 positive patients compared to PD-L1 negative patients, although the studies were not powered to detect a significant difference between these groups.

### Chemotherapy

Aside from trials limited to patients with collecting duct histology, there were 2 studies of traditional chemotherapy: one with carboplatin and paclitaxel and the second using capecitabine [34, 35]. Of the 16 patients who received carboplatin and paclitaxel, there was only 1 documented response to treatment, which was a CR in the patient with collecting duct histology. The trial of capecitabine in 51 patients with nccRCC had an ORR of 26%, including 2 patients with CR. Median PFS was 10.1 months (95% CI 8.7–11.5) and median OS was 18.3 months (95% CI 15.5–21.1) with a 1 year overall survival rate of 71%.

## TREATMENT OF nccRCC SUBTYPES

### Papillary nccRCC

A total of 7 studies, including 1 RCT and 6 single-arm phase II trials, included only patients with papillary histology, and an additional 15 studies of mixed histology reported results for papillary patients alone. The majority of the papillary-specific studies investigated the use of c-MET inhibition, due to the increased incidence of alterations in the *MET* proto-oncogene in these tumors [36]. Agents investigated included single agent savolitinib [37], foretinib [38], tivantinib [13], and crizotinib [39], as well as combination therapy with savolitinib plus durvalumab [40] and tivantinib plus erlotinib [13]. Tumor responses were mixed, ranging from an ORR of 0% for both tivantinib alone and tivantinib plus erlotinib [13], to an ORR of 27% for durvalumab plus savolitinib [40].

Three of these trials also included response rates stratified by the presence or absence of an alteration in the *MET* gene. Although the definition of “MET-altered” varied across trials, all found an increased ORR in patients with *MET* alterations compared to those without. In patients treated with savolitinib, all of the observed responses were in patients with *MET*-driven tumors with an ORR of 18% in this subgroup [37], while patients with a germline mutation in *MET* also had an improved response to foretinib compared to those without a mutation (ORR 50% vs. 9%) [38]. Additionally, in a trial of crizotinib, *MET*-altered patients had an ORR of 50% and 2 year OS rate of 75%, compared to an ORR of 6% and 2 year OS rate of 36.9% for wild-type patients [39].

As previously described, the ASPEN and ESPN trials each compared everolimus versus sunitinib in the first line setting [11, 12]. The overall trial results favoring sunitinib remained consistent for patients with papillary histology, with an ORR of 24% for sunitinib and 5% for everolimus in the ASPEN trial in this subset [12]. Use of sunitinib was also associated with longer PFS and OS compared with everolimus, when reported.

The RAPTOR and SUPAP trials evaluated everolimus and sunitinib respectively, in single arm trials of patients with type 1 and type 2 papillary RCC [41, 42]. Both trials showed modest activity in this subset [42]. Full results are summarized in Table 2.

### Chromophobe nccRCC

There were no studies that exclusively enrolled patients with chromophobe histology, however the ASPEN and ESPN trials included results for the subgroup of chromophobe patients. Contrary to the overall results, the median PFS was longer in the everolimus group than the sunitinib group in both trials, with a median PFS of 11.4 months for everolimus and 5.5 months for sunitinib in the ASPEN trial, and not reached for everolimus and 8.9 months for sunitinib in the ESPN trial (both non-significant). Two trials involving ICIs reported response rates for chromophobe patients alone. The ORR of pembrolizumab was 9.5% in chromophobe patients in Keynote-427 [30]; and the ORR of atezolizumab plus bevacizumab was 10% [33].

In studies of targeted therapies, chromophobe patients had comparable responses compared to all nccRCC patients with everolimus (ORR 29% vs. 10%; PFS 13.1 months vs. 5.2 months; OS 21.6 months vs. 14.0 months) [27], everolimus plus bevacizumab (ORR 40% vs. 29%) [28], axitinib (ORR 25% vs. 38%; PFS 11.0 months vs. 7.4 months; OS 22.2 months vs. 12.1 months) [25], and pazopanib (ORR 33% vs. 28%; PFS 18.3 months vs. 16.5 months; OS 18.9 months vs. NR) [26].

### Collecting duct nccRCC

Two single-arm phase II trials enrolled only patients with collecting duct histology. One study of gemcitabine plus cisplatin or carboplatin had a median PFS of 7.1 months (95% CI 3–11.3) and median OS of 10.5 months (95% CI 3.8–17.1) with an ORR of 26%, including 1 patient with a CR [43]. A similar trial of the combination of gemcitabine, cisplatin, and sorafenib reported a median PFS of 8.8 months (95% CI 6.7–10.9) and median OS of 12.5 months (95% CI 9.6–15.4) with an ORR of 30.8% [44]. Additionally, one trial of sunitinib reported results for collecting duct patients alone, with an ORR of 0% and median PFS of 3.1 months (95% CI 1.4–NR) [21].

## DISCUSSION

The total evidence base to guide treatment for patients with locally advanced or metastatic nccRCC remains limited and many questions regarding the optimal therapeutic strategy in this population are still unanswered. To our knowledge, there is only one prior systematic review and meta-analysis comparing the effectiveness and toxicities of systemic therapies for nccRCC [45] and a second review and meta-analysis comparing the efficacy of targeted therapies between ccRCC and nccRCC [46]. Given this limited evidence base, current clinical practice RCC guidelines from the European Association of Urology (EAU) and National Comprehensive Cancer Network (NCCN) recommend treatment based on limited data, and randomized studies using newer agents are desperately needed for this patient population.

Recently, the EAU RCC Guideline Panel decided to recommend sunitinib over everolimus and temsirolimus for first-line treatment of nccRCC based on a meta-analysis trend toward increased PFS favoring sunitinib over everolimus, although this did not reach statistical significance [45]. NCCN guidelines similarly categorize sunitinib as a “preferred regimen” for nccRCC, while everolimus is an “other recommended regimen,” and temsirolimus is a category 1 recommendation for patients in the poor-prognosis risk group but category 2A for other risk groups [47]. Our results support these general guidelines but also highlight differences in therapeutic strategies and treatment response across histologic subtypes of nccRCC [11, 12, 19, 21, 23-28, 33]. Trials in nccRCC continue to lump this diverse subgroup of cancers together, when the underlying biology and treatment efficacy clearly differs by subtype.

Additionally, newer strategies show promise in the upfront management of nccRCC, but comparative studies are lacking. Most notably, immune checkpoint inhibitors, alone or in combination, appear to have activity in papillary and unclassified RCC. With the need for additional high-level evidence to support treatment decisions, enrollment in clinical trials should be considered a preferred option for management of all patients with nccRCC. There are a number of ongoing trials in this setting, including a study of nivolumab plus cabozantinib (NCT03635892) and a study of lenvatinib plus everolimus in nccRCC (NCT02915783). These trials, among others, will hopefully provide further insight regarding optimal nccRCC management in the near future. Treatments with documented activity in larger promising phase 2 trials, such as pembrolizumab and the combination of atezolizumab plus bevacizumab, should be incorporated into guidelines to guide treatment choices given the lack of other effective agents and randomized trials. Additionally, combination regimens such as pembrolizumab plus axitinib have distinct rationale for use in nccRCC as well, given the modest activity of both checkpoint inhibitors and VEGF TKIs as monotherapy, although prospective data to support use of combination therapy is lacking.

### Papillary

Papillary RCC (pRCC) is the most common subtype of nccRCC and it is therefore possible to draw some conclusions from subgroup analyses and subtype-specific trials in pRCC. The highest level of evidence for treatment comes from the ASPEN and ESPN trials, both of which found that sunitinib is the preferred first-line treatment over everolimus based on a numerically superior OS and PFS [11, 12].

Recently, there has been an increased focus on genetic and molecular drivers of pRCC. Two such drivers are alterations in *MET*, which are found in 17–33% of type 1 papillary and 7% of type 2 pRCCs [48], and mutations in the gene for fumurate hydratase, which result in the familial syndrome of Hereditary Leiomyomatosis and Renal Cell Cancer (HLRCC) that is associated with an aggressive variant of type 2 pRCC. Our review found the results of trials using *MET* inhibitors to be somewhat underwhelming for unselected patients with pRCC, but ORR for patients harboring *MET* mutations are as high as 50% and further study of biomarker-selected patients is needed [37-40]. Cabozantinib, an inhibitor of multiple tyrosine kinases including c-MET and VEGFR2, has demonstrated efficacy in metastatic ccRCC [49, 50], but as of yet there are no published prospective studies evaluating its efficacy in nccRCC. However, retrospective studies suggest that it also has activity in nccRCC, with observed ORRs ranging from 27% [51] to 35% [52], including 1 patient with papillary RCC that achieved a CR [51]. The PAPMET trial (NCT02761057) comparing cabozantinib, crizotinib, savolitinib, or sunitinib in patients with metastatic papillary RCC is nearing completion of accrual and analysis of this study will hopefully provide additional evidence regarding the use of *MET* inhibitors in this population.

An additional study that did not meet criteria for inclusion in this review utilized bevacizumab plus erlotinib in patients with either HLRCC or sporadic pRCC. Patients with HLRCC had particularly robust response to this regimen with an ORR of 60% and PFS of 24.2 months, compared with an ORR of 29% and PFS of 7.4 months in patients with sporadic pRCC [53]. These targeted therapies appear promising within a select population, but genetic and molecular sequencing will need to be more widely used in order to appropriately identify patients that may benefit.

Finally, ICIs with or without TKIs are now standard of care for metastatic ccRCC, and our results suggest that this therapeutic approach has activity in papillary RCC as well, with ORRs of 28%, 25%, and 27% for pembrolizumab, atezolizumab plus bevacizumab, and durvalumab plus savolitinib, respectively [30, 33, 40]. However, survival data from these studies are not yet mature and it remains unknown if there is any benefit to combination therapies over single agent ICI.

### Chromophobe

Chromophobe RCC is typically a more indolent subtype of RCC with a lower risk of tumor progression or metastasis and longer cancer-specific survival. However, patients who do progress with locally advanced or metastatic disease have poor outcomes [54]. Our results suggest that there is at least modest efficacy in chromophobe RCC with VEGF TKIs, including sunitinib, axitinib, and pazopanib, and mTOR inhibition with everolimus or everolimus plus bevacizumab, and therefore these represent reasonable first-line treatment options. Since the ASPEN and ESPN trials both suggested a numerically longer median PFS with everolimus compared with sunitinib, this could be considered a standard at this point. Few chromophobe patients have been included in trials of ICIs thus far, but based on the two trials reported in this review, immune checkpoint inhibition may have limited efficacy in this subgroup [30, 33].

### Rare subtypes of nccRCC

Collecting duct carcinoma remains a rare but aggressive variant of nccRCC. A commonly utilized treatment for treatment is platinum-based chemotherapy, such as gemcitabine plus cisplatin or carboplatin. There are a handful of case reports describing patients with collecting duct carcinoma who responded to either cabozantinib [55], sunitinib [56], or sorafenib [57], however there are no prospective studies supporting the use of these therapies outside of a clinical trial setting. Our results support first-line use of chemotherapy and confirm the limited efficacy of TKIs in patients with collecting duct carcinoma. There were no studies of renal medullary carcinoma or translocation RCC that met criteria for inclusion in this review.

In the future, additional prospective studies enrolling nccRCC patients are required to further elucidate optimal treatment strategies and sequencing. Given the small number of patients with this disease, collaborative multi-institutional efforts are needed to provide the statistical power necessary to perform subgroup analyses based on patient and tumor factors. In particular, this review highlights a number of differences in treatment response between nccRCC histologies. Additional investigation will be required to determine whether these apparent differences may be related to differing efficacy of the treatment, inherent differences in tumor behavior, or differences in other patient-level characteristics. As our understanding of the molecular and genetic basis of nccRCC continues to improve, more studies will be needed to develop consensus definitions of clinically relevant mutations and to assess the prognostic and predictive value of existing and novel biomarkers.

One strength of this study is our review of the data stratified by histologic subtype. As previously mentioned, there can be significant variability in response between tumor histology and description of these differences is important. This study also includes review of 4 new trials utilizing ICIs, which is an area of growing interest and potential promise. A limitation of our study is the inability to perform a meta-analysis. As a systematic review, we are limited to population level rather than patient level data, and the significant heterogeneity of this population precluded pooling of results. Additionally, the majority of the studies included were single-arm phase II trials and expanded access programs, which are a less rigorous source of evidence than RCTs. This review focused on the efficacy of different therapies for nccRCC and as such does not include data regarding toxicity or quality of life for patients undergoing these treatments. However, as has been previously reported [45], the toxicities experienced by nccRCC patients are typically not different from those experienced by ccRCC patients receiving the same medications and are generally well-recognized class effects of each therapy. Despite these limitations, this study provides a valuable synthesis of the existing literature and highlights the need for ongoing efforts in this disease.

## CONCLUSIONS

This systematic review supports current consensus guidelines recommending sunitinib or enrollment in a clinical trial as first-line treatment options for nccRCC, but also suggests a more nuanced approach to management and new options for therapy such as immune checkpoint inhibition. All patients with locally advanced or metastatic nccRCC should have genetic and molecular sequencing to identify those that may benefit from targeted therapies.

## Supplementary Material

Supplemental Tables

## Figures and Tables

**Table 1:** Study characteristics and summary of outcomes of the included randomized controlled trials

Study	Comparator	Line	Total *n*	NCC n (%)	mOS, months (95% CI)	OS HR (95% CI)	mPFS, months (95% CI)	PFS HR (95% CI)	ORR (%)
ASPEN	Everolimus	First	57	57 (100)	13.2 (9.7 – 37.9)	1.12 (0.7 – 2.1)	5.6 (80% CI 5.5–6.0)	1.41 (80% CI 1.03–1.92)	9%
	Sunitinib	First	51	51 (100)	31.5 (14.8 – NR)		8.3 (80% CI 5.8–11.4)		18%
RECORD-3	Everolimus	First	238	31 (13)	–	–	5.1 (2.6–7.9)	1.5 (0.9–2.8)	–
	Sunitinib	First	233	35 (15)	–		7.2 (5.4–13.8)		–
ESPN	Everolimus	First	35	35 (100)	14.9 (8.0 – 23.4)	–	4.1 (2.7–10.5)	–	3%
	Sunitinib	First	33	33 (100)	16.2 (14.2 – NR)		6.1 (4.2–9.4)		9%
ARCC	Interferon-α	First	207	36 (17)	4.3 (3.2 – 7.3)		1.8 (1.6–2.1)		8%
	Temsirolimus	First	209	37 (18)	11.6 (8.9–14.5)	0.49 (0.29 – 0.85)	7.0 (3.9–8.9)	0.38 (0.23–0.62)	5%
SWOG 1107	Tivantinib	First Second	25	25 (100)	10.3 (7.3 – 15.7)	–	2.0 (1.8–3.0)	–	0%
	Tivantinib + Erlotinib	First Second	25	25 (100)	11.3 (6.7–21.9)		3.9 (1.8 – 7.3)		0%

Legend: (−) = Data not reported; CR = complete response; HR = hazard ratio; NCC = non-clear cell renal cell carcinoma; NR = not reached; mOS = median overall survival; mPFS = median progression-free survival; ORR = objective response rate.

**Table 2:** Study characteristics and summary of outcomes of trials in patients with papillary RCC. For trials that include patients with other histologies, only outcomes for the papillary patients are reported

Author	Year	Treatment	Line	Total n	Papillary n (%)	mOS, months (95% CI)	mPFS, months (95% CI)	ORR (%)
**VEGF TKIs**								
Park et al.	2018	Axitinib	Second or later	40	26 (65)	8.3 (4.1–12.5)	3.5 (0–10.9)	38%
Jung et al.	2018	Pazopanib	Any	29	19 (66)	NR	17.3 (14.8–19.8)	39%
Stadler et al.	2010	Sorafenib	Any	2504	107 (4)	–	–	3%
Armstrong et al.	2016	Sunitinib	First	51	33 (65)	–	8.1 (80% CI 5.8–11)	24%
Lee et al.	2012	Sunitinib	Any	31	22 (71)	–	–	36%
Molina et al.	2012	Sunitinib	Any	23	8 (35)	–	5.6 (1.4–7.1)	–
Procopio et al.	2008	Sorafenib	Second or later	136	15 (11)	–	–	7%
Ravaud et al.	2015	Sunitinib	First	61	61 (100)	Type 1:17.8 (5.7–26.1) Type 2:12.4 (8.2–16)	Type 1:6.6 (2.8–14.8) Type 2:5.5 (3.8–7.1)	Type 1:13% Type 2:11%
Tannir et al.	2012	Sunitinib	First Second Third	57	27 (47)	12.6 (7.3–36.9)	1.6 (1.4–5.4)	0%
Tannir et al.	2016	Sunitinib	First	33	14 (42)	14.9 (7.1–22.7)	5.7 (1.4–19.8)	–
Twardowski et al.	2017	Tivantinib	First Second	25	25 (100)	10.3 (7.3–15.7)	2.0 (1.8–3.0)	0%
**Immune Checkpoint Inhibitors**								
Suarez et al.	2019	Pembrolizumab	First	165	118 (71)	–	–	28%
**MET inhibitors**								
Schoffski et al.	2017	Crizotinib	Any	23	23 (100)	30.5 (12.3–NR)	5.8 (2.6–30.5)	17%
Choueiri et al.	2013	Foretinib	First Second	74	74 (100)	NR	9.3 (6.9–12.9)	14%
Choueiri et al.	2017	Savolitinib	Any	109	109 (100)	–	–	7%
**mTOR inhibitors**								
Armstrong et al.	2016	Everolimus	First	57	37 (65)	–	5.5 (80% CI 4.4–5.6)	5%
Escudier et al.	2016	Everolimus	First	88	88 (100)	21.4 (15.4–28.4)	4.1 (3.6–5.5)	1%
Koh et al.	2012	Everolimus	Any	49	29 (60)	10.9	3.4	7%
Tannir et al.	2016	Everolimus	First	35	13 (37)	16.6 (5.9–NR)	4.1 (1.5–7.4)	–
Dutcher et al.	2009	Temsirolimus	First	209	25 (12)	10.9 (7.8–15.1)	5.9 (3.7–9.0)	–
**Chemotherapy**								
Bylow et al.	2009	Carboplatin+Paclitaxel	First	17	16 (94)	–	–	0%
**Other/Combination Therapies**								
McKay et al.	2019	Atezolizumab+Bevacizumab	Any	65	12 (18)	–	–	25%
Powles et al.	2019	Durvalumab+Savolitinib	Any	41	41 (100)	NR	5.3 (1.5–12.0)	27%
Voss et al.	2016	Everolimus+Bevacizumab	First	35	4 (11)	–	13.8 (1.4–NR)	25%
Dutcher et al.	2009	Interferon-α	First	207	30 (14)	5.7 (4.3–7.8)	2.1 (1.8–4.3)	–
Twardowski et al.	2017	Tivantinib+Erlotinib	First Second	25	25 (100)	11.3 (6.7–21.9)	3.9 (1.8–7.3)	0%

Legend: (−) = Data not reported; CR = complete response; HR = hazard ratio; NE = not evaluable; NR = not reached; mOS = median overall survival; mPFS = median progression-free survival; ORR = objective response rate.

**Table 3:** Study characteristics and summary of outcomes of trials in patients with chromophobe histology. For trials that include patients with other histologies, only outcomes for the chromophobe patients are reported

Author	Year	Intervention	Line	Total n	Chromophobe n (%)	mOS, months (95% CI)	mPFS, (95% CI)	ORR (%)
**VEGF TKIs**								
Park, I	2018	Axitinib	Second or later	40	4 (10)	22.2 (−)	11.0 (−)	25%
Jung, K	2018	Pazopanib	Any	29	3 (10)	18.9 (−)	18.3 (11.9–24.7)	33%
Procopio, G	2008	Sorafenib	Second or later	136	3 (2)	–	–	0%
Stadler, W	2010	Sorafenib	Any	2504	20 (1)	–	–	5%
Armstrong, A	2016	Sunitinib	First	51	10 (19.6)	–	5.5 (80% CI 3.2–19.7)	10%
Lee, J	2012	Sunitinib	Any	31	3 (10)	–	–	33%
Tannir, N	2012	Sunitinib	First Second Third	57	5 (9)	–	12.7 (8.5–NR)	40%
Tannir, N	2016	Sunitinib	First	33	6 (18)	31.6 (14.2–NR)	8.9 (2.9–20.1)	–
**Immune Checkpoint Inhibitors**								
Suarez, C	2019	Pembrolizumab	First	165	21 (13)	–	–	10%
**mTOR inhibitors**								
Armstrong, A	2016	Everolimus	First	57	6 (10.5)	–	11.4 (80% CI 5.7–19.4)	33%
Koh, Y	2012	Everolimus	Any	49	8 (16)	21.6	13.1	29%
Tannir, N	2016	Everolimus	First	35	6 (17)	25.1 (4.7–NR)	NR	–
**Other/Combination Therapies**								
McKay, R	2019	Atezolizumab+Bevacizumab	Any	65	10 (15)	–	–	10%
Voss, M	2016	Everolimus+Bevacizumab	First	35	5 (14)	–	NR (1.9–NR)	40%

Legend: (−) = Data not reported; CR = complete response; HR = hazard ratio; NR = not reached; mOS = median overall survival; mPFS = median progression-free survival; ORR = objective response rate.

**Table 4:** Study characteristics and summary of outcomes of trials in patients with collecting duct histology

Author	Year	Intervention	Line	Total n	CD n (%)	OS, months (95% CI)	PFS, months (95% CI)	ORR (%)
Oudard, S	2007	Gemcitabine + Cisplatin or Carboplatin	First	23	23 (100)	10.5 (3.8–17.1)	7.1 (3.0–11.3)	26%
Sheng, X	2018	Gemcitabine + Cisplatin + Sorafenib	Any	26	26 (100)	12.5 (9.6–15.4)	8.8 (6.7–10.9)	31%
Tannir, N	2012	Sunitinib	First Second Third	57	6 (11)	–	3.1 (1.4–NR)	0%

Legend: (−) = Data not reported; CD = collecting duct; CR = complete response; HR = hazard ratio; NR = not reached; mOS = overall survival; mPFS = progression-free survival; ORR = objective response rate.
